# Percutaneous Microwave Ablation Versus Open Surgical Resection for Colorectal Cancer Liver Metastasis

**DOI:** 10.3389/fonc.2021.638165

**Published:** 2021-05-11

**Authors:** Qinxian Zhao, Zhigang Cheng, Zhiyu Han, Fangyi Liu, Xiaoling Yu, Xianliang Tan, Bin Han, Jianping Dou, Jie Yu, Ping Liang

**Affiliations:** ^1^ People’s Liberation Army General Hospital, Beijing, China; ^2^ Central People’s Hospital of Zhanjiang, Zhanjiang, China; ^3^ Affiliated Hospital of Shanxi College of Traditional Chinese Medicine, Taiyuan, China

**Keywords:** colorectal cancer, liver metastasis, ablation, resection, ultrasound

## Abstract

**Purpose:**

To compare the therapeutic outcomes between open surgical resection (OSR) and percutaneous microwave ablation (PMWA) for colorectal liver metastasis (CRLM) ≤3 cm.

**Methods:**

In this retrospective study, 200 consecutive patients with 306 CRLMs were reviewed. Overall survival (OS), disease-free survival (DFS), local tumour progression (LTP), intrahepatic distant recurrence, and extrahepatic metastasis were analysed to compare the therapeutic efficacy. Cox proportional hazards regression analysis was used to identify the prognostic factors for OS and DFS. Major complications and postoperative hospital stay were also assessed.

**Result:**

The 1-, 3-, and 5-year OS rates were 91.6%, 64.1%, and 46.3%, respectively, in the PMWA group and 89.7%, 62.4% and 44.7%, respectively, in the OSR group (P=0.839). The 1-, 3-, and 5-year DFS rates were 61.9%, 44.8%, and 41.3%, respectively, in the PMWA group and 58.1%, 24.4%, and 18.3%, respectively, in the OSR group (P =0.066). The two groups had comparable 5-year cumulative rates of intrahepatic distant recurrence (P=0.627) and extrahepatic metastasis (P=0.884). The 5-year cumulative LTP rate was lower in the OSR group than in the PMWA group (P=0.023). The rate of major complications was higher in the OSR group than in the PMWA group (P =0.025), and the length of hospital stay after treatment was shorter in the PMWA group (P<0.001).

**Conclusion:**

There were no significant differences in OS or DFS between the two groups. PMWA was associated with increased LTP, fewer postoperative days and fewer major complications.

## Introduction

Colorectal cancer (CRC) ranks the third most common type of malignancy in incidence and second in mortality (1). Disease burden is still increasing because of metastasis. Approximately half of all metastases that occur in the liver are either synchronous or metachronous (2–4). As the gold standard treatment for colorectal liver metastasis (CRLM), surgery indeed prolongs the overall survival (OS) of patients, with 5-year survival rates of up to 51% (5). However, concurrent medical conditions (multiple tumours located in different lobes or in a deep position, cardiopulmonary failure, older patients with hepatic or renal insufficiency, and debilitated patients after radiotherapy or chemotherapy) limit its widespread use, and only 10%-15% of patients with CRLM are eligible for open surgical resection (OSR) (6).

Other curative therapeutic modalities have developed, among which ablation is gaining focus because of its easy accessibility, high reproducibility, and minimal invasiveness (7–9). Several communities have performed comparative studies on the effectiveness and safety of radiofrequency ablation (RFA) and resection. The results showed that RFA and resection had similar OS rates for patients with CRLM ≤3 cm, and the recurrence rate was higher in patients treated with RFA than in patients treated with resection. The complication rate was lower for RFA. The outcome also demonstrated that recurrence was the dominant factor restricting the popularization of RFA.

Microwave ablation (MWA) has emerged as an innovative ablative technology (4, 10, 11) and has several advantages, including few needle insertions, extended ablation within a short time, sharp margins, minimal damage to the normal liver parenchyma, fast ablation times, large tumour ablation volumes, and consistently high and homogeneous temperatures, which are attributed to its working principle: tissue coagulation necrosis caused by the oscillation of polar molecular, non-reliance on electrical conductivity, less affected by the presence of blood vessels (12, 13). Furthermore, the forte of a percutaneous ultrasound (US)-guided modality virtually fulfils minimal invasiveness, and the ablation boundary can be timely adjusted and comprehensively evaluated.

MWA has been widely adopted for the treatment of hepatocellular carcinoma (14, 15). Some researchers have also explored its therapeutic effect on liver metastasis: Eng OS et al (16) showed that intraoperative MWA was a safe and effective treatment for patients with CRLM in tumours as large as 5.5 cm in size. Si et al. (17) researched 137 CRLM patients with 411 lesions (mean diameter 15.4±7.2 mm, range 5–67 mm) and concluded that US-guided PMWA was expected to become a routine method for the local tumour control of CRLM. However, the distinction of clinical outcomes between PMWA and OSR is still unclear. Thus, we conducted this study for patients with CRLM (maximum diameter CRLM ≤3 cm and number ≤3) to compare the clinical outcomes of the two modalities.

## Materials and Methods

### Study Design

This retrospective comparative study was approved by our ethical committee. The covariates were selected based on clinical relevance and the results of previous studies (18–21). Standardized terminology and reporting criteria (22) were used for the comparison of treatment outcomes between the two groups.

**Figure 1:**
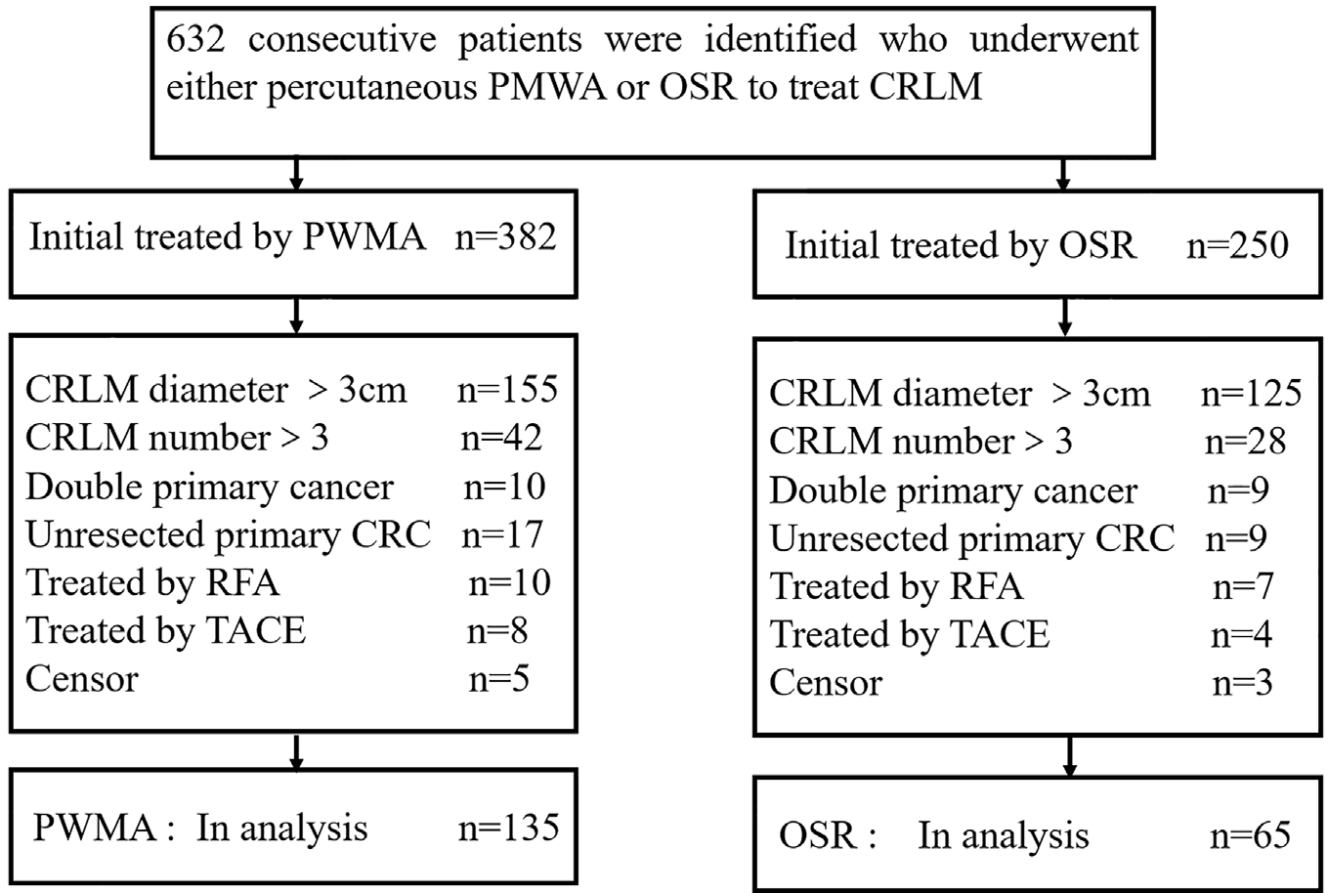
Flowchart of the study population. ORS: open surgical resection; PMWA: percutaneous microwave ablation; CRC: colorectal cancer.

All patients with curable CRLM who received either percutaneous MWA or OSR as the initial local treatment according to medical records between January 2009 and December 2017 were reviewed. The inclusion criteria were the maximum diameter of CRLM≤3 cm and the number of CRLM≤3. The exclusion criteria were as follows: (a) combined with another primary cancer; (b) treated with transcatheter arterial chemoembolization (TACE) or RFA; (c) unresected primary colorectal cancer; (d) missing the survival status. We identified 632 consecutive patients who underwent either percutaneous MWA (n = 382) or hepatic resection (HR) (n = 250) to treat CRLM. Based on these criteria, 432 patients were excluded: 280 with a CRLM size > 3 cm, 70 with a CRLM number > 3, 19 with another primary cancer, 17 treated with TACE, 12 treated with RFA, 26 with unresected primary colorectal cancer, and 8 with censored information. Two hundred patients (mean age, 56 years; range, 25–83 years) met the inclusion criteria and were included in our study. The screening flowchart for study patients is shown in **Figure 1**. A total of 135 patients (67.5%; 135 of 200 patients) with 213 lesions (79 patients had a single tumour, 56 patients had more than one tumour) underwent PMWA, and 65 patients (32.5%; 65 of 200 patients) with 93 lesions (41 patients had a single tumour, and 24 patients had more than one tumour) underwent OSR. The size and number of tumours were determined by two junior doctors through contrast enhanced MRI. For patients who cannot undertake MRI examination, contrast enhanced CT and contrast enhanced ultrasound were applied unsteadily.

A total of 94 patients (72%) in the PMWA group and 45 patients (69%) in the OSR group underwent comparable preoperative chemotherapy within 3 months before ablation or resection, and all patients underwent postoperative chemotherapy. The regular systemic chemotherapy regimen (FOLFOX (oxaliplatin, leucovorin, and 5-fluorouracil), XELOX (oxaliplatin, capecitabine), FOLFIRI (irinotecan, leucovorin, and 5-fluorouracil), or a combination of biologic-targeted agents (bevacizumab or cetuximab)) was carefully selectively according to the NCCN guideline presented in 2018 (23).

Every patient’s fundamental information and clinical variables used for the analysis of treatment outcomes included age, sex, body mass index (BMI), primary colorectal cancer location, comorbidities, maximal diameter, number of CRLM lesions, date and site of recurrence, cell differentiation, lymphatic invasion of primary colorectal cancer and status of the last follow-up. The patients were routinely screened with pretreatment examinations, such as laboratory tests (preoperative carcinoembryonic antigen (CEA), chest radiography, abdominal US, abdominal contrast material-enhanced computed tomography (CT) and/or pelvis magnetic resonance (MR) imaging) to obtain a comprehensive assessment of the situation before treatment. The diagnosis of CRLM was confirmed by pathologic findings in all patients after surgery in the OSR group. For all patients in the PMWA group, CRLM was verified by means of a percutaneous biopsy or based on more than two typical imaging demonstrations.

Local tumour progression (LTP) was defined as the discovery of tumour enhancement at the ablated area or resection margin of the hepatectomy. The presence of a tumour remote from the ablation or resection lesion within the liver was defined as intrahepatic recurrence. Extrahepatic metastasis was defined as the appearance of a tumour in the extrahepatic organs. Disease-free survival (DFS) was calculated from the date of treatment to the date of tumour recurrence or death or the last follow-up. OS was calculated from the date of local hepatic treatment to the date of death or the last follow-up.

### PMWA Procedure

All US-guided PMWA procedures were carried out by one of two experienced interventional radiologists (P.L., 20 years of experience in MWA; X.L.Y., 20 years of experience in MWA). Local anaesthesia was injected from the designated site in the skin after thorough disinfection. Biopsies was performed with an automatic biopsy gun under US guidance. A microwave antenna (15-gauge, 1.9 mm external diameter, 18 cm long) with a cooled-shaft system (KY-2000, Kangyou Medical Instruments, Nanjing, China) was then placed via the same skin incision. The microwave unit (KY-2000; Kangyou Medical, Nanjing, China) was capable of producing 100 W of power at 2450 MHz. General anaesthesia was administered after all insertions via the peripheral vein. A power output of 50 W for 10 minutes was routinely used. During ablation, the adjacent organ and coverage of the hyperechoic area were monitored in real time. If the heat-generated hyperechoic water vapour did not encompass the entire tumour, prolonged microwave emission was applied until the desired temperature was reached. The needle track was cauterized when withdrawing the needle to avoid the possible seeding of tumour cells.

### OSR Technique

The operation was performed by one of two experienced surgeons (C.Y.L. and J.K., both with more than 15 years of experience in hepatobiliary surgery). Tumour burden, liver remnant, and the possibility of a negative resection margin were evaluated before the operation with an imaging technique, which is a reference for the surgical approach. The removal of one Couinaud segment containing both the tumour and corresponding hepatic parenchyma was defined as anatomic resection. Nonanatomic resection (NAR) refers to removal of the tumour with a minimal tumour-free margin, and wedge resection and tumour enucleation were excluded (24, 25). The pattern of resection was selected according to the proposed guidelines (26). All surgical procedures were performed by using standard surgical techniques for hepatectomy (27). All resection specimens were evaluated by a conventional histopathologic examination after the operation.

### Follow-Up

The primary colorectal cancer of the patients in both groups was excised before local hepatic therapy. Contrast material-enhanced US or MR was applied after PMWA or OSR to determine the technical success of the procedure. Technique effectiveness was evaluated by the absence of enhancement of any ablated areas at the follow-up with a contrast-enhanced examination performed 1 month after MWA. The absence of microscopic tumour invasion at the resection margin was the gold standard used to assess the resection margins in the OSR group. Postoperative surveillance included a physical examination, laboratory tests (routine blood test, CEA) and contrast-enhanced ultrasound or MR scans were performed every 3 to 6 months for the first 3 years and annually thereafter to diagnose the first recurrence.

### Statistical Analysis

Demographic and preoperative data from the two groups were compared by using Student’s t test for continuous variables, with the assumption of normality of the two samples, and the Pearson x2 test or Fisher’s exact tests for categorical variables by using SPSS 21 software (SPSS, Chicago, Ill). Missing values were filled in by using multiple imputation method. DFS and OS were calculated by using the Kaplan-Meier method, and the differences were assessed to compare the long-term therapeutic outcomes between the two groups by log-rank tests. A Cox proportional hazards model was used to test for significant factors on survival when the variables were significantly different at P≤0.05 in the log-rank test with a univariate or multivariate analysis.

## Results

### Patient and Tumour Characteristics

In total, 200 consecutive patients who fulfilled the inclusion criteria were enrolled. A total of 135 patients were treated with PMWA, and the OSR group comprised 65 patients. Eighteen patients (28%) in the hepatectomy group were women. Forty-seven patients (35%) in the ablation group were women. Patients in the two groups had comparable characteristics, including age, sex, BMI, smoking status, alcohol consumption, CEA level, cell differentiation, comorbidities, maximal tumour diameter, primary colorectal cancer location, pattern of metastasis, and number of CRLM lesions. The median diameter of CRLM was 2.1 cm (range, 0.7 cm to 3.0 cm) in the ablation group and 2.2 cm (range, 0.8 cm to 3.0 cm) in the hepatectomy group. The baseline patient and tumour characteristics are presented in **Table 1**. More than one CRLM was observed in 56 patients (41%) in the ablation group and in 24 patients (37%) in the hepatectomy group. Eighty CRLMs (59%) originated from the colon in the ablation group, and 50 (77%) originated from the colon in the hepatectomy group.

**Table 1:** Baseline characteristics of study patients and tumors.

Parameter*	MWA Group (n = 135)	SR Group (n = 65)	P Value
**Age (y)†**	57.1(25-83)	55.2(35-82)	0.247
**No. of men**	88(65.2)	47(72.3)	0.314
**BMI**	23.7(15.6-34.9)	24.0(18.2-31.1)	0.243
**Preoperative CEA(ng/ml) at diagnosis of liver metastasis**	26.0(0.3-369.8)	26.4(0.27-188.1)	0.968
**No. of Smoking**	53(39.3)	26(40)	0.819
**No. of Alcohol consumption**	60(44.5)	26(40)	0.644
**Tumor characteristics**			
**Number of liver metastatic tumor**	1.6(1-3)	1.5(1-3)	0.645
**Liver metastatic maximum diameter (cm)**	2.1(0.7-3)	2.2(0.8-3)	0.104
**Location of primary colorectal**			
** colon**	79(58.5)	42(64.6)	0.409
** rectum**	56(41.5)	23(35.4)	
**Histological differentiation**			
** High grade**	8(5.9)	2(3.1)	0.632
** Medium grade**	100(70.1)	42(64.6)	
** Low grade**	24(17.8)	13(20)	
**No. of Comorbidities**	41(30.4)	22(33.8)	0.131
**Synchronous Metastasis**	65(48.1)	41(63.1)	0.067
**Positive LN at staging of primary disease**	87(64.4)	41(63.1)	0.374
**Preoperative chemotherapy within 3 months**			
** Yes**	98(72.6)	45(69.2)	0.622
** No**	37(27.4)	20(30.8)	

Unless otherwise indicated, data are number of patients, with percentages in parentheses. BMI, Body Mass Index; CEA, carcinoembryonic antigen; LN, lymph node. ^†^Data are medians, with the range in parentheses. *Represent that the variable were selected based on clinical relevance.

The mean follow-up period was 33.2 months (range, 2.0–102.0 months) for PMWA and 39.4 months (range, 6.0–116.0 months) for OSR (P =0.209). A 100% technique effectiveness was achieved in both groups (PMWA: 135 of 135 treatments; OSR: 65 of 65 treatments) according to the follow-up CT or MR scan after four weeks of local hepatic treatment.

Forty-five patients died (33.3%; 45 of 135 patients) in the PMWA group, and 24 patients died (36.9%; 24 of 65 patients) in the resection group. The causes of death were tumour progression (colorectal local and systemic progression), multiple organ failure and non-tumour-related disease (**Table 2**).

**Table 2:** Cause of death during of follow-up.

Variable	PMWA	OSR
Tumor progressive	41	20
Respiratory failure	2	2
Pulmonary infection	1	0
Pulmonary encephalopathy	1	0
Hepatic encephalopathy	0	1
Septic shock	0	1
Treatment-related	0	0

Data are numbers of patients.

### Survival and Subgroup Analysis

**Figure 2:**
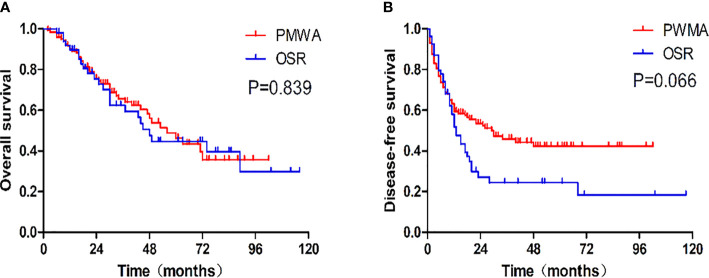
**(A)** Graph shows cumulative overall survival rates after each treatment. The 1-, 3-, and 5-year overall survival rates were, respectively, 91.6%, 64.1%, and 46.3% in the PMWA group and 89.7%, 62.4%, and 44.7% in the OSR group. **(B)** Graph shows cumulative disease-free survival rates after each treatment. The 1-, 3-, and 5-year disease survival rates of the PMWA group were, respectively, 61.9%, 44.8%, and 41.3% in the PMWA group and58.1%, 24.4%, and 18.3% in the OSR group. PMWA, percutaneous microwave ablation; OSR, open surgical resection.

**Figure 3:**
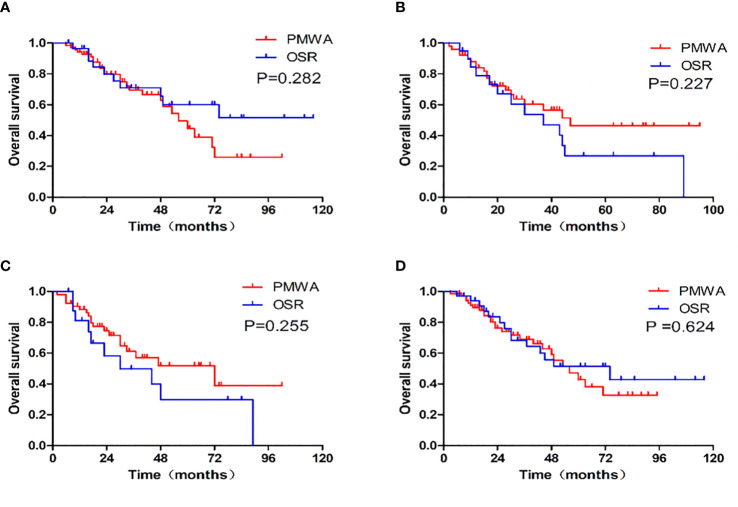
Graphs show the overall survival and survival curves after stratification between patients with single tumor (P=0.282), more than one tumor (P=0.227), older than 60(P=0.255), younger than 60 (P=0.624). **(A)**: single CRLM; **(B)**: more than one CRLM; **(C)**: patients older than 60, **(D)**: patients younger than 60; PMWA, percutaneous microwave ablation. OSR, open surgical resection.

There was no statistically significant difference in OS (P=0.839) or DFS (P = 0.066) between the two groups. The 1-, 3-, and 5-year OS rates were 91.6%, 64.1%, and 46.3%, respectively, in the PMWA group and 89.7%, 62.4%, and 44.7%, respectively, in the OSR group. For DFS, the 1-, 3-, and 5-year cumulative survival rates were 61.9%, 44.8%, and 41.3%, respectively, in the PMWA group and 58.1%, 24.4%, and 18.3%, respectively, in the OSR group (**Figure 2**). There was no significant difference in the cumulative OS rate between the two groups after stratification for patients older than 60 years (P=0.255), younger than 60 years (P=0.624), with a single tumour (P=0.282), or with more than one tumour (P=0.227) (**Figure 3**). All the survival P values were based on Kaplan-Meier analyses for the entire curves.

### The Comparison of Recurrence Between the Two Groups

**Figure 4:**
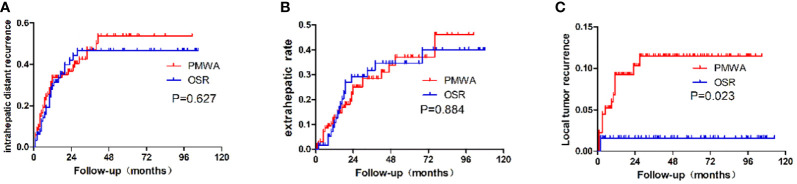
Recurrence patterns after PMWA or OSR for colorectal liver metastasis. **(A)**: Intrahepatic distant recurrence; **(B)**: Extrahepatic recurrence; **(C)**: local tumor recurrence; PMWA, Radiofrequency ablation; OSR, open surgical resection; CRLM, Colorectal liver metastasis.

The type of recurrence was different between the groups. The 5-year cumulative local recurrence rate was higher in the PMWA group than in the OSR group (P=0.023). However, there was no significant difference in the 5-year cumulative rates of intrahepatic distant recurrence (P=0.627) or extrahepatic metastasis (P=0.884) of the two groups. The mean time to first recurrence (first appearance of LTP, intrahepatic distant recurrence, or extrahepatic metastasis) was 22.6 months in the PMWA group and 23.4 months in the OSR group; 71 patients (53%; 71 of 135 patients) after PMWA and 64 patients (58%; 38 of 65 patients) after OSR developed recurrence, including local, intrahepatic, and systemic recurrence (**Figure 4**).

### Univariate and Multivariate Analysis

Significant predictors were further assessed with a multivariable Cox proportional hazards model after evaluation by the univariate analysis when P<0.05. Of all the variables, BMI (P = 0.041; HR, 0.935; 95% CI: 0.877, 0.997), CEA level (P = 0.017; HR, 1.004; 95% CI: 1.002, 1.007), number of tumours (P =0.018; HR, 1.425; 95% CI: 1.061, 1.913), lymph node metastasis (P = 0.027; HR, 2.079; 95% CI: 1.087, 3.978), and preoperative chemotherapy (P = 0.010; HR, 0.536; 95% CI: 0.333, 0.863) were found to be significant risk factors affecting OS in the univariate analysis. Multivariate Cox proportional hazards regression analyses showed that the number of tumours (P = 0.027; HR, 1.471; 95% CI: 1.044, 2.072) and preoperative chemotherapy (P =0.033; HR, 0.545; 95% CI: 0.313, 0.951) were independent prognostic factors for OS (**Table 3**). Regarding DFS, the number of tumours (P = 0.040; HR, 1.303; 95% CI: 1.013, 1.677) and comorbidities (P =0.040; HR, 1.499; 95% CI: 1.019, 2.204) were significant predictors in the univariate analysis. After multivariate Cox proportional hazards regression analyses, the number of tumours (P = 0.044; HR, 1.300; 95% CI: 1.012, 1.670) was identified as an independent prognostic factor (**Table 4**).

**Table 3:** Cox Survival Analysis of Predictors OS in the Whole Population.

Characteristic	Univariate Analysis			Multivariate Analysis		
	HR	95%CI	P Value	HR	95%CI	P Value
**Age**	1.013	0.991-1.035	0.255			
**BMI**	0.935	0.877-0.997	0.041	0.938	0.874-1.007	0.077
**Preoperative CEA(ng/ml) at diagnosis of liver metastasis**	1.004	1.0021-1.007	0.017	1.003	0.999-1.006	0.145
**Number of liver metastatic tumor**	1.425	1.061-1.913	0.018	1.471	1.044-2.072	0.027
**Liver metastatic maximum diameter (cm)**	1.371	0.896-2.097	0.146			
**Gender**	1.071	0.641-1.789	0.794			
**Smoking**	1.044	0.738-1.475	0.809			
**Alcohol consumption**	1.055	0.750-1.484	0.760			
**Comorbidities**	0.957	0.588-1.559	0.861			
**Metastasis type**	1.384	0.838-2.286	0.205			
**LN status at staging of primary disease**	2.079	1.087-3.978	0.027	1.586	0.799-3.147	0.187
**Preoperative chemotherapy within 3 months**	0.536	0.333-0.863	0.010	0.545	0.313-0.951	0.033
**Location of primary colorectal**	0.840	0.516-1.366	0.482			
**Histological differentiation**	1.523	0.972-2.385	0.066			
**Treatment type**	0.950	0.577-1.565	0.840			

**Table 4:** Cox Survival Analysis of Predictors DFS in the Whole Population.

Characteristic	Univariate Analysis			Multivariate Analysis		
	HR	95%CI	P Value	HR	95%CI	P Value
**Age**	0.991	0.975-1.008	0.316			
**BMI**	0.953	0.902-1.006	0.079			
**Preoperative CEA (ng/ml) at diagnosis of liver metastasis**	1.003	1.000-1.007	0.057			
**Number of liver metastatic tumor**	1.303	1.013-1.677	0.040	1.300	1.012-1.670	0.040
**Liver metastatic maximum diameter (cm)**	1.192	0.863-1.647	0.288			
**Gender**	0.871	0.585-1.297	0.496			
**Smoking**	0.846	0.648-1.105	0.220			
**Alcohol consumption**	0.850	0.665-1.085	0.192			
**Comorbidities**	1.499	1.019-2.204	0.040	0.675	0.455-1.001	0.05
**Metastasis type**	1.080	0.734-1.588	0.696			
**LN status at staging of primary disease**	1.424	0.905-2.240	0.127			
**Preoperative chemotherapy within 3 months**	1.174	0.774-1.780	0.450			
**Location of primary colorectal**	0.968	0.656-1.430	0.872			
**Histological differentiation**	0.933	0.641-1.360	0.720			
**Treatment type**	0.694	0.467-1.034	0.072			

### Major Complications and Posttreatment Hospital Stay

These results reveal that ablation is associated with lower rates of major complications (Clavien-Dindo classification grades 3-5) than surgical resection (1.5% vs 7.7%, respectively; P=0.025). There were no treatment-related deaths in either group. Two major complications were detected in the ablation group: one patient experienced embolus formation of the portal vein, and another patient developed a secondary infection after ablation. Unlike the ablation group, there were 2 hepatic abscesses (1 biliary leakage, 1 massive haemorrhage within the abdominal cavity) and 1 ulcer formation of the abdominal wall in the hepatectomy group. The mean postoperative hospital stay was three-fold lower in the ablation group than in the resection group (**Table 5**).

**Table 5:** Major Complications and Hospital Stay after Treatment.

outcome	PMWA (N=135)	OSR (N=65)	P value
Posttreatment hospital stay(mean, d)	3(1-7)	11(6-34)	< 0.001
Major complications	2	5	0.025
Embolus Formation of Portal Vein	1	0	
Secondary Infection	1	0	
Hepatic Abscesses	0	2	
Biliary Leakage	0	1	
Massive Hemorrhage	0	1	
Ulcer Formation of The Abdominal Wall	0	1	

## Discussion

Surgical resection is the gold standard treatment for liver metastasis (23). Percutaneous MWA is gaining popularity due to its short hospital stay and low morbidity and mortality rates and thus may be an alternative treatment for patients with CRLM. However, there is not enough evidence-based medicine to support the value of MWA in CRLM. The present study compared the two modalities in patients with CRLM≤3 cm and number ≤3.

In this retrospective study, no significant differences in therapeutic outcomes, including OS and DFS, were detected between the two groups. The OSR group had a higher rate of major complications and a longer postoperative hospital stay. The treatment modality affected neither OS nor DFS in the univariate and multivariate analyses.

Patient age was stratified to further analyse the clinical outcomes between the two groups. The results demonstrated that patients older than 60 years who underwent ablation had a better 5-year cumulative OS rate than those who underwent open surgery (38.9% vs. 29.9%, respectively; P=0.255), although there was no significant difference between the two groups. These age-dependent differences may originate from the fact that the traumas were fewer for treatment with ablation and that the time needed for rehabilitation was shorter after ablation than resection. In fact, all patients in the ORS group who developed major complications were more than 60 years old.

Preoperative chemotherapy was related to prolong OS in our study. All patients underwent postoperative chemotherapy (either PMWA or OSR), while some patients did not receive preoperative chemotherapy due to personal characteristics. Statistical results demonstrated that preoperative chemotherapy was a protective factor. Zhang et al. (28) researched 199 patients, including 318 CRLMs with a median follow-up of 30.2 months, and concluded that regular chemotherapy after MWA improved OS, which may be because chemotherapy can downsize colorectal metastases, inhibit the viability of tumour cells, increase the probability of the complete eradication or inactivation of tumours, reduce the recurrence rate and thus improve the survival rate.

The prognostic value of CEA has been confirmed in patients with CRLM by previous investigators (29, 30). CEA enhances cell aggregation and promotes cancer cell metastases, and the immune system is suppressed by the release of suppressor factors from normal lymphocytes induced by CEA (31). In the univariate analysis performed in the present research, the level of pretreatment CEA was found to be a positive prognostic indicator of OS. However, CEA was not found to be an independent factor in the multivariate analysis, which may be correlated with the limited sample size.

Previous studies have revealed that the LTP rate was 5%-13% (11, 32) for MWA compared to 3.7% (33) for resection. The LTP rate was higher in the PMWA group than in the OSR group (P=0.023) in the present study. In clinical practice, open surgery is more likely to eliminate microsatellite nodules to obtain an adequate margin because of its preponderance of peripheral lesions. However, repeated resection has the potential risk of major complications, such as intra-abdominal haemorrhage, because of local adhesion after previous hepatic resection, mostly in patients who underwent palliative treatment, such as chemotherapy, immunotherapy, targeted therapy or TACE.

It was difficult to achieve an ideal clearance circumferential margin for ablation, which included micrometastases and microvascular invasion around the tumour. In the early period, a 5-mm ablative margin was used according to the treatment of hepatocellular carcinoma in our centre. Hepatocellular carcinoma has a false capsule, and heat is more easily accumulated. However, liver metastasis has specific biological behaviours, including infiltration growth, no capsule and a clear boundary. An enlarged ablative margin is essential to reduce local recurrence according to a previous study (6, 34). Therefore, we expanded the ablative margin above 1 cm if the tumour was in a prime location (not adjacent to vital organs such as major blood vessels or bile ducts). Furthermore, PMWA has the advantage of high repeatability, and the size of the recurrent tumour is small because of regular follow-up; therefore, it is easy for PMWA to achieve complete ablation. LTP can be remedied in a timely manner in patients who undergo PMWA.

The current study has intrinsic defects because of its retrospective nonrandomized design, which may produce uncontrolled confounding effects. The statistical strength may have decreased and led to bias because of the small sample size of patients. The results might not be reproducible in other settings because of its single-centre trait. Despite these limitations, our findings may help validate the value of percutaneous MWA for patients with CRLM. However, a study with a larger number of patients and a longer follow-up evaluation is needed to further verify the value of MWA for its range of applications and its appropriateness to treat CRLM.

## Conclusion

This similar result suggests that PMWA is a favourable alternative and preferred choice for patients with small CRLM. Furthermore, it may be suitable for older (≥60 years) patients. Moreover, an enlarged ablation margin may be an effective method used to reduce local tumour recurrence.

## Data Availability Statement

The raw data supporting the conclusions of this article will be made available by the authors, without undue reservation.

## Ethics Statement

The studies involving human participants were reviewed and approved by Ethics Committee of Chinese PLA General Hospital.

## Author Contributions

Concept and design: PL, JY, JPD. Experiments and procedures: All authors. Writing of article: QXZ, JY. Statistical analysis: QXZ. All authors contributed to the article and approved the submitted version.

## Funding

This work was supported by Grants 81627803, 81971625, 91859201 and 81871374 from the National Scientific Foundation Committee of China, Grant JQ18021 from the National Scientific Foundation Committee of Beijing, Fostering Funds for National Distinguished Young Scholar Science Fund and the National Clinical Research Center for Geriatric Diseases (NCRCG-PLAGH-2019011) of Chinese PLA General Hospital, Grant 2018ZX10723-204 from the National Key R&D Program of Ministry of Science and Technology of China.

## Conflict of Interest

The authors declare that the research was conducted in the absence of any commercial or financial relationships that could be construed as a potential conflict of interest.
